# Supplementary material for: Supporting adolescents living with HIV within boarding schools in Kenya
Source: PLoS One. 2021 Dec 2;16(12):e0260278. doi: 10.1371/journal.pone.0260278 (PMC8638902; doi:10.1371/journal.pone.0260278)
Supplement: S1 Appendix — This file outlines components of RCP activities coordinated through the SHC concerning identification of LLHIV, linkages to, and retention in care within schools. The HIV-responsive school checklist outlines criteria consistent with characteristics of an HIV-responsive school. (DOCX) [file pone.0260278.s001.docx]

**S1 Annex**

**Components of RCP Activities within the SHC**

The following activities enable identification of LLHIV, linkages to, and retention in care within schools and are coordinated through the SHC. They include:

- Strengthening school and HCFs collaboration to ensure all LLHIV **keep** **appointments**, are retained in care, and maintain viral load suppression.
- Engagement and **capacity building of teachers and primary and secondary caregivers** on treatment literacy and support for LLHIV.
- Mainstreaming HIV and AIDS literacy and support activities in school health clubs to **facilitate stigma reduction**, increase knowledge on HIV, and foster positive attitudes towards LLHIV within the school environment.
- Establishing school-led systems and **structures for PSS**, including peer support and identifying treatment buddies.
- Increasing **access to social protection** **services** through referrals and linkage within and outside the school environment.

**Checklist for the characteristics of a HIV-responsive school:**

A school is certified in conjunction by MOH, MOE and RCP once they fulfil the below criteria.

- Appoint a focal point for LLHIV within the school (ASA).
- Respect the dignity, confidentiality and privacy of LLHIV during school admissions and beyond.
- Ensure confidentiality of the private information from parents/guardians of LLHIV during admission and beyond
- Support HIV prevention services and referral (e.g., HIV testing services, pre-exposure prophylaxis, post-exposure prophylaxis, gender-based violence services, etc.).
- Assist LLHIV in attending their clinic appointments.
- Ensure LLHIV can access psychosocial counselling.
- Support DOT when indicated.
- Designate a safe space for LLHIV to store drugs safely and confidentially.
- Support timely taking of the ARVs by LLHIV.
- Encourage LLHIV to disclose to trusted persons in school and have treatment buddies within boarding schools, as applicable, for ongoing PSS.
