# Supplementary material for: Supporting adolescents living with HIV within boarding schools in Kenya
Source: PLoS One. 2021 Dec 2;16(12):e0260278. doi: 10.1371/journal.pone.0260278 (PMC8638902; doi:10.1371/journal.pone.0260278)
Supplement: Attachment — Submitted filename: ENGAGING BOARDING SCHOOLS TO SUPPORT ADOLESCENTSLIVING WITH HIV IN KENYA.docx [file pone.0260278.s002.docx]

**ENGAGING BOARDING SCHOOLS TO SUPPORT ADOLESCENTSLIVING WITH HIV IN KENYA**

**REVIEWER COMMENTS:**

**KEY MESSAGE:**

- The study is an important one and will contribute to the knowledge gap regarding adolescents living with HIV in the boarding school environment.
- The initial impression is that the authors wished to evaluate the impact of the RCP intervention on retention in care and treatment. However, on further reading, the real question being asked is whether one can implement an intervention to improve student-school-HCF interactions in boarding secondary schools.
- The initial aim is therefore not addressed by the study findings
- I highlight what needs to be done in the various sections of the manuscript to improve its message and contribution to the scientific community.

A. **Introduction:**

- The question the authors sought to answer has changed from the introduction through the results and conclusions.
- On page 3 of the manuscript:

“The overarching goal of RCP is to increase linkages to and retention in care and treatment among AYLHIV aged 10-24 years. Implemented in coordination with the Kenya Ministry of Education (MOE) and Ministry of Health (MOH), the RCP school interventions included enhancing partnerships between boarding schools and RCP healthcare facilities (HCFs) while building capacity of schools to support AYLHIV.”

This does not seem to be captured in the results and conclusions of the manuscript. It however appears the main question was whether the intervention can be implemented.

***Suggestion for improvement:*** *Focus your introduction and aim both in abstract and main text on the intervention in the two counties and that is an offshoot of the main RCP school interventions.*

- The context of the study in the introduction is clear.

B. **Methods/Study design/Statistics:**

- Well done.
- Few concerns that if addressed will make it more readable.
- Waiver for consent was requested and granted because of the minimal risk intervention. Did you consider the psychological consequences of inadvertent disclosure for those in school without complete disclosure about their HIV status?
- **If so, it is not clear from the description how that was addressed during the study implementation.**
- What of the stigma related to classmates realizing their HIV status?
- **How was this mitigated against because it could have far reaching effects on the adolescent? If this was addressed, please outline how?**
- Also, it would help to know if all schools were selected or if not**, the method used in identifying the intervention schools.**
- You mention as a limitation that you did not have non-intervention schools to compare with**. Any reason why you did not consider intervention and non-intervention schools.**
- It is not clear how improved linkage between Schools and HCT was assessed. **In other words, if a child has a primary HCF from outside the catchment of the study, were they transferred to the county HCF?**
- **DOT:** How was the improvement in this assessed? **And how was it practically done?**
- **Counselors:** How was their improved skill/improved psychosocial support assessed?
- **Training curriculum for teachers:** You used the HCP APOC curriculum. This is not been purposed for secondary school teachers. **How did you modify it to enable the education given to the schools to be dependable?**

***Suggested improvements:*** *Explain each item above, if considered how it was addressed?*

- **Outcomes:** Your main outcomes seem to increased numbers of Secondary school-HCF units, increased AYLWH who were paired to HCF, number of teachers and counsellors trained on HIV care.
- I would have expected additional outcomes which were readily available to you to be presented in methods and results. These include adherence to medications, viral suppression (which comes in the discussion although missing in results), and psychological well-being using standard tools for assessment.

***Suggested improvement:*** *If you have contact of the individual AYLWH you can improve the manuscript by looking at baseline data on adherence to medications, viral suppression, and psychological well-being on these participants and including it.*

- **Outcome changes:** The increased numbers from school-HCF units, teachers, counselors etc were never subjected to inferential statistics.

***Suggested improvement:*** *If you have pre and post-intervention numbers, you some inferential statistics can be done. However, the best approach would have been using a non-intervention group.*

C. **Results, Tables and Figures:**

- -Overall impression: This is fairly well done. However some data is mentioned as percentages in the text but no raw number accompany that data.
- **Examples:**

“Overall, throughout RCP implementation, the number of schools with competent guidance and counselling teachers increased by **19.4%,** the number of schools with teachers trained on caring and supporting AYLHIV increased by **99%,** and the number of schools linked to a HCFs increased by **62%,** compared to baseline”.

***Suggestion for improvement:*** *The bolded numbers need to have raw number –for example from xx to xx (19.4%). Having some of the data in the tables and referring to them will improve readability.*

D. **Conclusions:**

- Indicates there is potential to improve retention in care and reduction of stigma. However, the results do not have any outcomes for any of the potential heath indicators.

***Suggested change:*** *Indicate the specific parameters that improved although the improvements were not tested statistically to confirm significance.*

E. **References and Appendices:**

- Fairly well done

Thank you.

Sincerely

W. Nyandiko
